# The Prognostic Role of the C-Reactive Protein and Serum Lactate Dehydrogenase in a Pediatric Series of Bone Ewing Sarcoma

**DOI:** 10.3390/cancers14133064

**Published:** 2022-06-22

**Authors:** Giada Del Baldo, Rachid Abbas, Maria Antonietta De Ioris, Valentina Di Ruscio, Iside Alessi, Evelina Miele, Angela Mastronuzzi, Giuseppe Maria Milano

**Affiliations:** 1 Department of Pediatric Hematology/Oncology, Bambino Gesù Children’s Hospital, IRCCS, Piazza di Sant’Onofrio 4, 00165 Rome, Italy; giada.delbaldo@opbg.net (G.D.B.); mantonietta.deioris@opbg.net (M.A.D.I.); valentina.diruscio@opbg.net (V.D.R.); iside.alessi@opbg.net (I.A.); evelina.miele@opbg.net (E.M.); angela.mastronuzzi@opbg.net (A.M.); 2 Centre de Recherche en Epidémiologie et Santé des Populations, INSERM, Université Paris-Sud, 94800 Villejuif, France; abbas.rachid@gmail.com

**Keywords:** Ewing sarcoma, prognostic factors, survival, biomarkers

## Abstract

**Simple Summary:**

Ewing sarcoma is a rare and aggressive tumor of childhood and adolescence. Over the years, different prognostic factors have been explored to stratify high-risk patients. The roles of C-reactive protein (CRP) and serum lactate dehydrogenase (LDH) as potential new prognostic factors would be a useful and simple for risk stratification, but they have rarely been investigated. In our work, we analyze the role of LDH and CRP as prognostic factors in a population of pediatric and adolescent patients affected by Ewing sarcoma. Our study confirms the potential prognostic role of LDH at diagnosis as an independent prognostic factor. LDH evaluation is not expensive, and it can be beneficial for developing countries where diagnostic and staging resources in the pediatric oncology field are poor.

**Abstract:**

Background: Ewing sarcoma (ES) is a rare and aggressive pediatric cancer. Numerous studies have attempted to identify new prognostic biomarkers. The predictive value of serum LDH and CRP has not been clearly described, to date. Methods: The objective of our retrospective study was to investigate the prognostic value of LDH and CRP levels and their association with overall survival in a series of ES patients. Results: Between 2004 and 2019, 89 ES patients were included. In a univariable analysis, high levels of LDH and CRP were associated with the worst prognosis. In a multivariable analysis, only higher LDH values remained associated with a lower survival. The high-LDH-level group experienced all 21 deaths registered in our population (24%) and about 90% of disease progressions. The 5-year overall survival was 66.4% in the high-LDH-level group, while no deaths were observed in the low-LDH-level group. The 5-year progression-free survival was 57.9% in the high-LDH-level group versus 80.4% in the low-LDH-level group. Conclusions: In our study, LDH levels at diagnosis were strongly correlated with the prognosis, and they might be considered a prognostic factor in Ewing sarcoma. The LDH value, along with its very low cost and its reproducibility in almost all centers, make it suitable as a potential prognostic biomarker in clinical practice.

## 1. Introduction

The Ewing sarcoma (ES) is a highly malignant tumor formed from small-undifferentiated round cells in bone or soft tissue. ES is more common in children and adolescents, but it can occur at any age. From a molecular point of view, ES is specifically characterized by the presence of recurrent chromosomal translocations that involve chromosomes 11 and 22 in more than 85% of cases. The translocation occurs in a pathognomonic chimeric fusion gene, EWSR1/FLI1 [1]. In the last 30 years, the survival rate has slightly improved, reaching a 65% to 75% 5-year overall survival (OS) for patients with localized disease [2,3,4], but patients with metastases had a less than 30% 5-year OS [5]. Relapsed disease still represents a challenge due to its dismal prognosis and poor available treatment options [6,7,8,9]. Over the years, several predictors have been explored to stratify patients, including: genetic aberrations, age at diagnosis, tumor volume, site, histologic response, and bilateral and multiple pulmonary or bone marrow metastases [10,11,12,13,14,15]. Since then, many studies have attempted to identify factors that more accurately predict the clinical outcome, investigating new biomarkers, molecular targets, liquid biopsy, exosomes, and new radiological technologies [16]. The prognostic value of biomarkers such as C-reactive protein (CRP) and serum lactate dehydrogenase (LDH) are rarely investigated in ES, although these biomarkers are largely used, cheap, and well standardized. CRP, a non-specific marker of inflammation, can be increased in malignancies [17], and its role as a predictive biomarker of survival has been confirmed in certain adult cancers. In particular, a high level of CRP was associated with poor prognosis in several tumors, i.e., in renal-cell carcinoma, gastric cancer, lung cancer, breast cancer, and in bone sarcoma [18]. LDH is a pyridine-linked enzyme that catalyzes the reduction of free pyruvate to lactate during glycolysis, and the oxidation of l-lactate to pyruvate during gluconeogenesis [19]. Due to high levels of glucose utilization in cancers, LDH levels were found to be increased in hematological malignancies and some adult solid tumors [20,21,22,23,24,25]. Moreover, LDH is expressed extensively in body tissues and released during tissue injury in several diseases [26,27]. The serum level of LDH was found to be correlated with tumor burden, growth, survival and invasive potential [28]. Serum LDH has been proposed as a prognostic factor in EWS in certain studies, although conflicting results still remain [29]. Few data have been published to evaluate whether the levels of serum LDH may be a prognostic factor for survival in patients with ES, but the results reported are discordant and inconclusive [30]. CRP has also retained interest as a prognosis biomarker in soft-tissue sarcomas (STS) [31]. In this study, we evaluated the prognostic value of LDH and CRP at ES diagnosis for the association of biomarkers with survival.

## 2. Materials and Methods

At our hospital, one of the main pediatric oncology centers in Italy, a chart review of patients affected by Ewing’s sarcoma admitted between 1998 and 2020 was carried out. All patients had histologically and molecularly proven ES, as per ongoing protocols, and molecular studies available in that period. An experienced pathologist reviewed the histological diagnosis of all patients.

The patient data included: demographics, histology, radiologic and laboratoristic data, treatment details, status, and time at last follow-up.

An institutional review board approved the study, finalizing the exportation of data from hospital records. Written informed consent for the study was obtained from parents or legal guardians.

### 2.1. Biomarkers

Biomarker data collected in routine practice were used, including LDH and CRP measured at diagnosis, before treatment initiation. The center used an in vitro test for the quantitative determination of lactate dehydrogenase in serum and plasma, using cobas c systems, Roche. LDH reference values were: 135–214 UI/L for girls older than 15 years, 135–225 UI/L for boys older than 15 years and 135–214 UI/L for children (2–14 years). An immunoturbidimetric assay for in vitro quantitative determination of CRP in serum and plasma, using cobas c systems, Roche was used. CRP normal value was <0.5 mg/dL.

### 2.2. Treatment

Between 1998 and 2005, the ES chemotherapy protocol in use at our center was ICE/CAV induction [32]. Since 2005, localized or metastatic ESs were treated according to AIEOP (Italian Association of Pediatric Oncology and Hematology)/ISG (Italian Sarcoma Group) protocols EW-1 and EW-2, respectively. The chemotherapy consisted of vincristine, doxorubicin, ifosfamide, cyclophosphamide, and etoposide. Local treatment included surgery (S), radiotherapy (RT), or both. In poor responders (necrosis < 90% of tumor viable) or metastatic patients, high-dose chemotherapy (HDCT) combined with busulfan and melphalan (BU-MEL), and followed by autologous hematopoietic stem-cell transplantation (ASCT), were performed. Moreover, in the EW-2 protocol, a maintenance therapy, low-dose cyclophosphamide plus celecoxib, was started after HDCT and total lung irradiation (TLI) [33]. Informed consent for treatment was obtained from parents or legal guardians.

### 2.3. Statistical Analysis

All patients included in the study with at least one valid baseline measure of LDH or CRP were included in the descriptive analysis. The LDH and CRP measures were transformed to reduce right skewness with the natural logarithm and the natural logarithm +1, respectively. The log-transformed biomarkers were introduced separately in multivariable Cox models for OS adjustments of the following commonly reported prognostic factors of ES: age and metastatic status at diagnosis, location of the disease (axial versus peripheric), and intratumor necrosis. Each models’ assumptions, the log linearity of age and both biomarkers, and the proportional hazards assumption were assessed with supremum tests on martingale residuals and their transforms over time. We analyzed the discriminant ability of the biomarkers through follow-up with time-dependent ROC curves. For biomarkers associated with survival, a classification and regression tree (CART) model was used to find a cut-off with 10-fold cross-validation. Patients’ characteristics were compared via groups of biomarker levels (high and low levels at baseline) using chi-square, Fisher’s exact, and Student’s *t*-tests, as appropriate. OS was defined as the time from diagnosis to the date of last contact or death from any cause. Progression-free survival (PFS) was defined as the time from diagnosis to the date of disease progression, last contact, or death from any cause. The median follow-up was assessed using Schemper’s method. OS and PFS curves based on biomarker levels were derived from Kaplan–Meier estimates and compared with log-rank tests. All comparison tests were two-sided and considered significant at the 5% level.

## 3. Results

### 3.1. Clinicopathological Features

Since 1998, 127 ESs diagnosed and treated in our center, while 89 patients fit all the criteria and presented in this chart review.

The median age at diagnosis was 10 years (range from 1 to 24 years) with a balanced sex ratio (1.23 male to female). There were two patients older than 18. Twenty-four out of eighty-nine patients (27%) had metastatic disease at diagnosis. Fifty-one (57%) were good responders after neoadjuvant chemotherapy (necrosis > 90%). All patients had an LDH baseline measure with a median of 443 UI/L (IQR: 322–550) and a CRP baseline measure with a median of 0.80 mg/dL (IQR: 0.14–2.88). For detailed characteristics of the overall population, see Table 1. 

### 3.2. Treatment Details

Systemic chemotherapy based on the EW1 and EW2 protocols was performed in 50 patients (56%) and 23 (26%) patients, respectively. A combination of ICE (ifosfamide, carboplatin, and etoposide) plus CAV (cyclophosphamide, doxorubicin, and vincristine) regimens was given to 16 patients (18%).

Local treatment included surgery in 48 (54%) patients, surgery and radiotherapy in 21 (24%) patients, and only radiotherapy in 10 (11%) patients.

### 3.3. Evaluation of Prognostic Factors

In the univariate analysis, known prognostic factors (age at diagnosis, site, necrosis, presence of metastasis) and high levels of LDH and CRP were associated with the worst prognosis. While, in the multivariable analysis, higher LDH alone remained associated with poor prognosis and survival; one log increase in the baseline LDH was associated with a 58% (95% CI: 2–245%) increase in risk of death (Table 2). 

**Figure 1:**
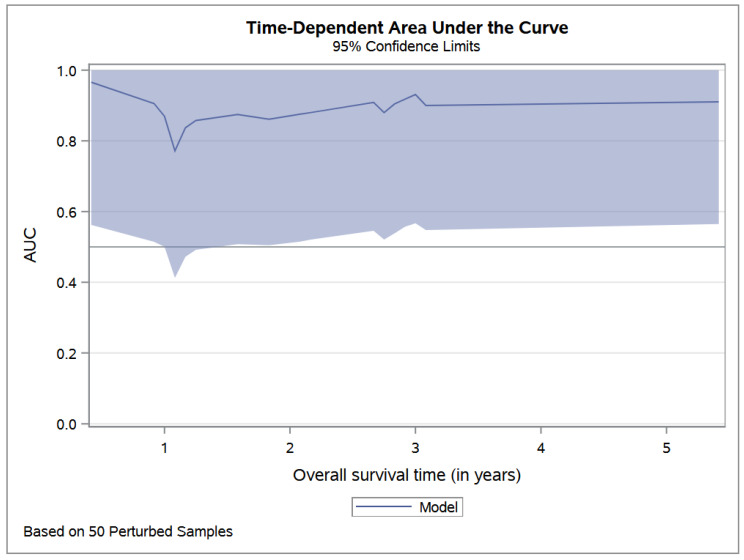
Time-dependent area under the ROC curve of overall survival multivariable model, including LDH.

The area under the ROC curve was constant over time (Figure 1). At 3 years, the addition of LDH to the multivariable model increased the area under the ROC curve from 0.89 to 0.92. 

Based on the CART model, the cross-validated threshold for log (LDH) was 5.809, corresponding to the value of 333 UI/L. Low levels of LDH refer to baseline values below 333 UI. According to this cut-off, patients were divided into the high LDH level (n = 27) and low LDH level (n = 62). Table 3 provides the patients’ characteristics according to the LDH level. No correlation was found between LDH levels and metastatic disease (*p*-value = 0.19).

CRP levels were significantly higher in patients with metastatic disease, with a median CRP level of 2.70 UI/L (*p*-value = 0.03). However, despite its power’s balancing with other prognostic factors, mainly metastatic status at diagnosis, the CRP did not have an independent prognostic value on survival: log(CRP) HR was 1.09 (95% CI: 0.63–1.89).

### 3.4. Survival

The median follow-up was 4.8 years. At the last follow-up, 21 (24%) deaths were observed, all of which were included in the high-LDL-level group. In addition, 32 (36%) disease progressions were observed, including 28 in the high-LDL-level group.

**Figure 2:**
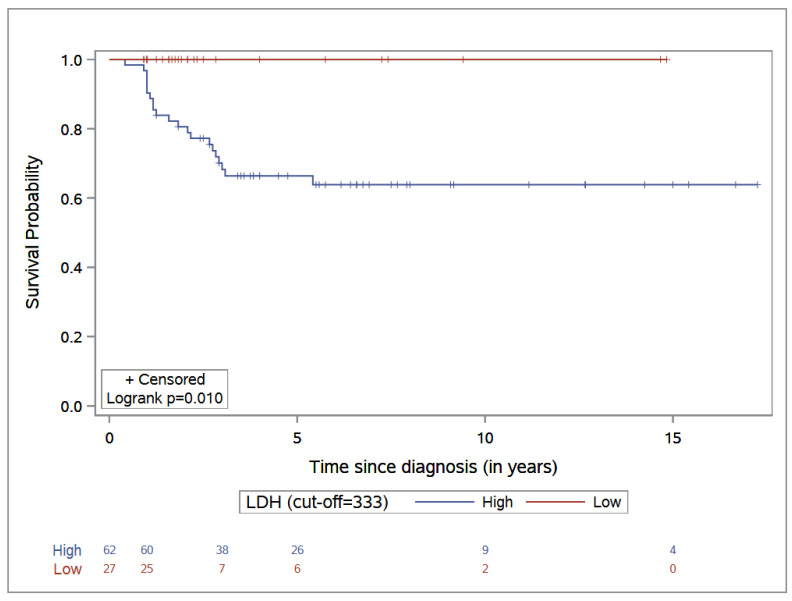
Overall survival stratified by LDH level.

The 5-year OS was 66.4% (95% CI: 53.6–77.1) in the high-LDL-level group, while no deaths were observed in the low-LDL-level group (log-rank test *p*-value = 0.01) (see Figure 2). 

**Figure 3:**
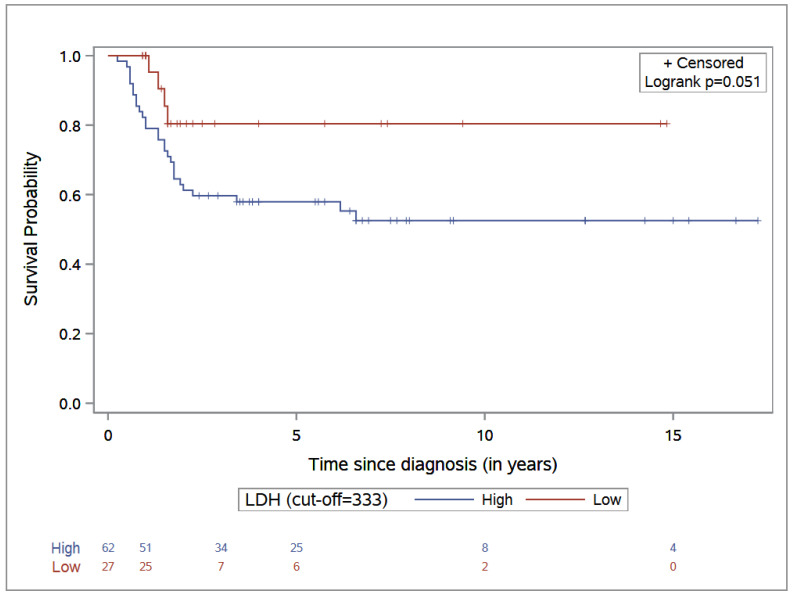
Progression-free survival stratified by LDH level.

The 5-year PFS was 57.9% (95% CI: 45.5–69.4) in the high-LDL-level group, versus 80.4% (95% CI: 59.1–92.1) in the low-LDL-level group (*p*-value = 0.05) (see Figure 3).

## 4. Discussion

ES is a rare and aggressive tumor of childhood and adolescence. Several studies have confirmed that prognostic factors such as age, site of disease, metastasis, and necrosis < or >90% are associated with survival [10,11,12,13,14,15]. In the same period, other studies explored LDH or CRP as potential new prognostic factors that would be useful in improving treatment stratification. 

High serum LDH may reflect a high tumor burden, and has been shown to be a poor prognostic factor in several reports since the late 1990s [34]; these variables have been the subject of animated debate from numerous scientific reviews that either validated or denied their role, even in sarcoma patients.

Firstly, Ferrari et al. reported an analysis of data concerning factors predicting the histological response of patients in a population of non-metastatic ES younger than 40 years, including LDH; they did not find a correlation with histological response such as fever, and the site and size of the tumor [35]. The same group, led by Bacci et al., proved that in several studies, LDH—together with gender, age, tumor volume, number of chemotherapy drug types, and histological response—were independent prognostic factors for disease-free survival in a series of 579 consecutive pediatric and adult ES cases [36]. They also investigated the prognostic relevance of serum LDH in 618 patients with a diagnosis of ES. Similarly to our study, they demonstrated that the percentage of patients with higher pre-treatment LDH levels was enhanced among metastatic patients, and was strongly correlated with lower OS and shorter TTP [37]. Impressively, they reported that prognosis worsened in patients with localized disease but higher LDH levels, assuming that a linear correlation could be detected among LDH levels and incidence of relapse [38].

A report from Biswas and colleagues confirmed, using a multivariate analysis, that raised LDH (as well as low hemoglobin level) adversely affected OS among extraosseous ES patients [39]. 

The first systematic review of tumor markers in ES was published in 2003, combining 84 papers investigating several biochemical markers, mostly the pretreatment LDH levels. Despite the non-optimal, clinically significant cut-off value for serum LDH ranges in different laboratories, the authors confirmed that patients with higher levels of serum LDH had an increased risk of tumor-related death and of disease recurrence, 2.9 times and 3.4 times greater, respectively, than for those with low value [40]. The lack of specificity and standardized cut-off values are the two main problems highlighted by the authors, and they obstruct the possibility of using LDH as a routinary marker in ES patients. 

As the scientific debate on this promising initial research has certainly not stopped, other clinical studies have yet to confirm these interesting results. Recently, Nakamura et al., who analyzed 142 adult patients with STS, did not replicate those results, and LDH was found to be a prognostic factor in neither the uni-nor the multivariate analysis [41]. In a small pediatric population of 20 patients with rhabdomyosarcoma, Bien et al. did not find a significant difference in LDH values compared with survival [23]. 

As nonreliable parameters have been established, different working groups have tried to investigate new potential diagnostic markers. An interesting study suggested a new classifier called Ces, using clinical laboratory indicators that should distinguish ES patients from healthy individuals, with a good ability to discriminate tumor metastasis and EFS [42].

Concerning the potential role of CRP, in this discussion, we should mention a meta-analysis performed by Wang at al., from which the author concluded that the correlation between pre-treatment levels of CRP and prognosis was concrete in patients’ sarcomas [43]. The same results were then reproduced in 332 adult patients with STS by Nakamura et al., who demonstrated that high CRP level is related to decreased disease-specific survival and local recurrence-free rate [44]. Moreover, J Szkandera et al. described a correlation between pre-operative plasma CRP levels and a poor clinical outcome of cancer-specific survival, disease-free survival, and OS in 304 adult STS patients [31]. 

As we discussed above, there are relatively few contradictory data, demonstrating there are insufficient results to support, or to not support, the predictive value of CRP and LDH.

One reason could consist in the heterogeneity of the population studied and in the methods used to determine the impact of these variables in prognosis. In this light, we started this chart review focused on identifying what would be the target value of both LDH and CRP outside of the laboratory range values.

Currently, our study is the first conducted in a pediatric and adolescents series of ES cases that has investigated LDH and CRP as prognostic factors. Using univariate analysis, we confirmed, as is already known, that the presence of metastasis, age at diagnosis, and necrosis < 90% were associated with the worst prognosis. Meanwhile LDH showed an independent prognostic factor impact on survival in both the overall and progression-free curves. Moreover, we underlined that the LDH measures were made before starting therapies and validated in a single institutional laboratory, and no patient had another chronic illness might justify increased LDH values. We found an association between CRP and metastasis at diagnosis. After adjusting using the multivariable model, CRP was not an independent prognostic factor on survival. 

In contrast to previous studies, in which the LDH threshold was chosen based on the laboratory reference, we derived an LDH threshold based on our population of interest, using a CART model to find a cut-off with 10-fold cross-validation. This approach allows for a better prognostic stratification of patients with ES, and better tailoring of clinical strategies according to prognostic characteristics. We believe that our threshold based on a 10-fold cross-validation procedure is robust to sample bias and could be generalizable to other settings.

At a median follow-up of 4.8 years, 24% of ES patients had died, all of whom were included in the high-level-LDH group. Moreover, 32 patients (36%) presented disease progression, and 28 of them were included in the high-LDL-level group. Unexpectedly, CRP did not show significant prognostic value in our multivariable analysis.

Our study, as with others before, tries to contribute to the debate on how LDH and CRP have potential roles in ES patients. We confirmed that LDH segregates population risk. We extrapolated the LDH mean value independently of the arbitrary division referred to the internal laboratory values using a CART model, which confirmed the prognostic impact of this in the EW population. The CRP value was correlated with prognosis in neither the uni-nor the multivariate analysis; this may be due to the non-specificity of this marker to tumor growth. In fact, it is often expressed in several inflammatory conditions or comorbidities simultaneously present in oncology patients.

Beyond these results, although not the primary scope of our study, LDH values might be taken in consideration for promising approaches specifically targeting LDH. In fact, Yeung et al. demonstrated that in ES-derived cell lines and the intravenous administration of LDH inhibitors resulted intratumoral drug accumulation, inducing cell death and suddenly reducing tumor growth [45]. These preliminary results demonstrate a potential role of LDH inhibitors as anti-cancer agents, and monitoring LDH values might help clinicians to identify patients who would benefit more from this application.

Our study limitations are the retrospective design and the small sample size, even though it is the largest study reported in pediatric ES that confirms LDH prognostic value at diagnosis.

## 5. Conclusions

In conclusion, our work highlights the potential prognostic role of LDH at diagnosis as an independent prognostic marker. Moreover, LDH evaluation is not expensive, and is included in routine laboratory assessment worldwide. Certainly, it can be beneficial for developing countries where diagnostic and staging resources in the pediatric oncology field are poor. Furthermore, monitoring LDH values could also have therapeutic implications, by using LDH inhibitors as a new antineoplastic agents in patients with higher plasmatic values. However, further large-scale and prospective studies are needed to confirm this statement. 

## Figures and Tables

**Table 1:** Clinical characteristics of the overall population.

Characteristics at Diagnostic	Overall Population	n = 89
Age at diagnosis (years)	Mean (sd)	10.7 (4.3)
	Median [Q1–Q3]	10 [8–14]
	Minimum/Maximum	1/24
Gender	Male, n (%)	49 (55%)
	Female, n (%)	40 (45%)
Tumor localization	**Peripheral site n (%)**	**53 (60%)**
	Lower limb, n (% of peripheral)	21 (40%)
	Upper limb, n (% of peripheral)	13 (24%)
	Rib bone, n (% of peripheral)	19 (36%)
	**Axial site, n (%)**	**36 (40%)**
	Vertebra and spinal column, n (% of axial)	13 (36%)
	Skull, n (% of axial)	9 (25%)
	Pelvis, n (% of axial)	13 (36%)
	Sacrum, n (% of axial)	1 (3%)
Tumor stage at diagnosis	Metastatic, n (%)	24 (27%)
Tumor necrosis > 90%	Presence, n (%)	51 (57%)
Local treatment option	Surgery, n (%)	48 (54%)
	Radiotherapy, n (%)	10 (11%)
	Radiotherapy and surgery	21 (24%)
	No local treatment	10 (11%)
Systemic treatment	ICE plus CAV	16 (18%)
	AIEOP/ISG EW 1	50 (56%)
	AIEOP/ISG EW 2	23 (26%)
LDH (UI/L)	Median [Q1–Q3]	443 [322–550]
LDH (UI/L) in M+ (n = 24)	Median [Q1–Q3]	455 [341–651]
LDH (UI/L) in localized (n = 65)	Median [Q1–Q3]	423 [312–528]
CRP (mg/dL)	Median [Q1–Q3]	0.8 [0.14–2.88]
CRP (UI/L) in M+ (n = 24)	Median [Q1–Q3]	2.70 [0.54–5.82]
CRP (UI/L) in localized (n = 65)	Median [Q1–Q3]	0.50 [0.14–2.07]

**Table 2:** Multivariate survival model with LDH as a continuous predictor (per known prognostic factor).

	Univariate Analysis		Multivariate Analysis *	
	HR (95% CI)	*p*-Value	HR (95% CI)	*p*-Value
LDH (log scale)	4.52 (2.08–9.83)	<0.0001	1.58 (1.02–2.45)	0.04
Age at diagnosis	1.01 (1.00–1.02)	0.01	1.01 (1.00–1.02)	0.01
Necrosis	3.39 (1.24–9.31)	0.02	1.56 (0.49–4.95)	0.45
Axial tumor	1.56 (0.66–3.69)	0.31	1.35 (0.50–3.68)	0.56

* Multivariate Cox model stratified by metastatic status at diagnosis, adjusted on LDH; age at diagnosis intratumoral necrosis; and tumor localization (axial versus peripheric).

**Table 3:** Characteristics of patients according to the level of LDH at diagnosis.

		TOTAL (n = 89)	LDH Low * (n = 27)	LDH High * (n = 62)	*p*-Value
Age at diagnosis (in years)	Median [Q1–Q3]	10 [8–14]	12 [10–14]	9.5 [8–13]	0.13
	Min/Max	12/288	24/288	12/228	
Sex	Male, n (%)	49 (55.1%)	15 (55.6%)	34 (54.8%)	0.95
Status at diagnosis	Peripheral localization, n (%)	53 (59.6%)	14 (51.9%)	39 (62.9%)	0.36
	Metastases, n (%)	24 (27.0%)	6 (22.2%)	18 (29.0%)	0.51
Necrosis	n (%)	51 (57.3%)	17 (63.0%)	34 (54.8%)	0.48
Local treatment	Surgery, n (%)	48 (53.9%)	15 (55.6%)	33 (53.2%)	0.84
	Radiotherapy, n (%)	32 (36.0%)	11 (40.7%)	21 (33.9%)	0.54
CRP	Median [Q1–Q3]	0.8 [0.1–2.9]	0.5 [0.1–1.8]	1.0 [0.2–3.5]	0.25
	Min/Max	0.1/23.4	0.1/13.1	0.1/23.4	

* LDH cut-off = 333 UI/L.

## Data Availability

The data presented in this study are available in this article.
